# Clinical findings in two cases of atypical scrapie in sheep: a case report

**DOI:** 10.1186/1746-6148-3-2

**Published:** 2007-02-13

**Authors:** Timm Konold, Andrew Davis, Gemma Bone, John Bracegirdle, Sharon Everitt, Melanie Chaplin, Ginny C Saunders, Saira Cawthraw, Marion M Simmons

**Affiliations:** 1 Neuropathology, Veterinary Laboratories Agency Weybridge, New Haw, Addlestone, UK; 2 Royal Veterinary College, Population Biology and Disease Control Research Group, North Mymms, Hatfield, UK; 3 State Veterinary Service Cardiff, Llandrindod Wells, Powys, UK; 4 TSE Molecular Biology 4, Veterinary Laboratories Agency Weybridge, New Haw, Addlestone, UK

## Abstract

**Background:**

Atypical scrapie is a recently recognised form of transmissible spongiform encephalopathy of sheep that differs from classical scrapie in its neuropathological and biochemical features. Most cases are detected in apparently healthy sheep and information on the clinical presentation is limited.

**Case presentation:**

This report describes the clinical findings in two sheep notified as scrapie suspects and confirmed as atypical scrapie cases by immunohistochemistry and Western immunoblotting. Although both sheep displayed signs suggestive of a cerebellar dysfunction there was considerable variation in the individual clinical signs, which were similar to classical scrapie.

**Conclusion:**

Any sheep presenting with neurological gait deficits should be assessed more closely for other behavioural, neurological and physical signs associated with scrapie and their presence should lead to the suspicion of scrapie.

## Background

Scrapie is a transmissible spongiform encephalopathy (TSE) of small ruminants, which has been reported in many countries of the world. There is considerable variation in the clinical presentation of individual cases but the major clinical signs are behavioural, sensory and locomotor changes [1]. These include increased anxiety, teeth grinding, pruritus, a crouched or wide-based stance, ataxia and hypermetria. In addition, tremors and loss of weight or bodily condition are frequently displayed. Pruritus resulting in alopecia is most frequently observed, and the "nibble reflex" where sheep respond to scratching of the dorsum with characteristic lip and tongue movements, is often used as a test for pruritus [2]. (See Additional file 1: Movie 1 as an example of a positive scratch test in classical scrapie). Neuropathologically, the disease is characterised by the presence of vacuoles in neurons and neuropil, together with accumulations of disease-associated prion protein (PrP^sc^) in the brain. For confirmatory diagnosis, the histopathological and immunohistochemical (IHC) examination of a brain section at the level of the obex, which must include the dorsal motor nucleus of the vagal nerve, is usually sufficient. More recently, rapid post-mortem tests are used for scrapie surveillance, which are based on detection of the proteinase-resistant part of PrP^sc^, PrP^res ^[3].

Susceptibility of sheep to scrapie is associated with polymorphisms which affect codons 136, 154 and 171 in the PrP gene, which encodes the prion protein. The allele with valine (V) at codon 136, arginine (R) at codon 154 and glutamine (Q) at codon 171 (VRQ) are linked to increased susceptibility, whilst the allele with alanine (A) at codon 136 and arginine at codons 154 and 171 (ARR) are linked to increased resistance. Sheep carrying the VRQ/VRQ or ARQ/VRQ genotype are most susceptible to scrapie and sheep of the ARR/ARR genotype are most resistant [4].

In 2003, an unusual type of scrapie in sheep, named Nor98, was described in Norway [5]. Affected sheep displayed predominantly ataxia in the absence of pruritus, and neuropathological findings were mainly restricted to the cerebellar and cerebral cortices, whilst vacuolation and PrP^sc ^accumulation in the brainstem at the level of the obex was sparse or absent. Western immunoblot analysis showed a PrP^res ^glycoprofile with a protein band of lower molecular mass than in previous scrapie cases. Similar "atypical" scrapie cases have since been found in many other countries [6-12] and mainly in sheep of PrP genotypes not usually associated with "classical" scrapie. In addition, the presence of an ARQ allele carrying a phenylalanine (F) residue at codon 141 (AF^141^RQ) and ARR or AHQ PrP alleles appeared to be associated with atypical scrapie [13,14]. A transmission study of atypical scrapie to transgenic mice expressing ovine PrP confirmed the infectious nature of the disease and also suggested that the atypical cases were caused by a unique strain [15].

The majority of these atypical cases were detected by active scrapie surveillance in [apparently] healthy slaughtered animals or in fallen stock, which implies that they either displayed no clinical signs or presenting signs were ones that are not usually associated with scrapie. The clinical information available on atypical cases reported as clinical suspects is very limited and only based on observations by farmers. The purpose of this report is to describe the clinical signs in two atypical scrapie cases reported as clinical suspects in the United Kingdom (UK) based on a clinical and neurological examination conducted at the Veterinary Laboratories Agency Weybridge. This may aid in the recognition and reporting of atypical scrapie suspects in countries where scrapie is a notifiable disease.

## Case presentation

### History

Both sheep (05/377 and 06/008) were 5-year-old Welsh Mountain ewes, which belonged to the same farm and were born and raised in a flock of approximately 650 sheep of various ages. Both cases had an AHQ/AF^141^RQ PrP genotype as determined by DNA sequencing of the PrP gene coding region [14]. They were reported by the owner as clinical suspects in October 2005 and January 2006, respectively. Scrapie cases had not been found previously on this farm.

### Clinical findings

#### CASE 05/377

Clinical signs observed by the farmer and inspecting state veterinary officer were tremors, ataxia, changes in temperament and loss of bodily condition. Ataxia had been the first clinical sign noticed.

The physical examination revealed a heart rate of 110 bpm, a rectal temperature of 39.2°C and reduced rumen contractions. The bodily condition was good (body condition score [BCS] 3 out of a maximum of 5). Abnormal neurological findings were nervous behaviour when approached or handled, reduced withdrawal reflexes on all four limbs, delayed hind limb repositioning when the limb was abducted and hind limb ataxia with hypermetria (see Additional file 2: Movie 2 showing hypermetria). Cranial nerve deficits were not present. There was a small area of alopecia on the left hip region, minor lacerations at the lateral aspects of both hocks and several small scabs on the bridge of the nose. Scratching of the thoracolumbar dorsum elicited repeatable rhythmical head and body movements; these were inconsistently displayed further caudal on the dorsum.

Rumination was observed during a passive observation for 15 minutes; significant abnormalities other than a single episode of teeth grinding were not detected.

#### CASE 06/008

Gait abnormalities and postural changes were seen by the farmer and state veterinary officer. Loss of bodily condition had been the first observed clinical sign according to the owner.

The physical examination revealed a heart rate of 92 bpm, a rectal temperature of 37.9°C and normal rumen contractions (3 contractions/2 min). The bodily condition was poor (BCS 1).

Abnormal neurological findings were a fine head tremor, which was more pronounced when the animal was blindfolded, a wide-based stance of the hind limbs and hind limb ataxia with a crouching posture on turns (see Additional file 3: Movie 3 showing ataxia and crouching). Repositioning of the hind limbs was delayed when the limbs were abducted or the animal pushed to one side. In addition, the menace response was deficient although other cranial nerve lesions were not evident (normal palpebral and pupillary light reflex) and the ocular fundus appeared normal. (See Additional file 4: Movie 4 showing an absent menace response and normal palpebral reflex).

Alopecia was not observed, although there were several papules on the bridge of the nose, and shorter, stained wool on the left shoulder, which may have been the result of rubbing. The sheep did not respond to scratching (see Additional file 5: Movie 5 showing no response to scratching).

The sheep ruminated temporarily during a passive observation for 15 minutes but appeared dull compared to its pen mate, with a low head carriage or the head resting on the floor and remained lying down when the pen was entered, which was considered abnormal.

### Postmortem diagnosis

**Figure 1:**
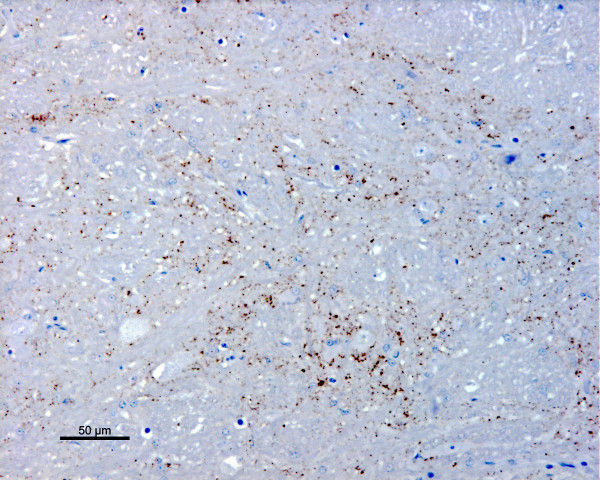
**PrP^sc ^immunolabelling in the spinal tract nucleus of the trigeminal nerve (case 06/008)**. Fine granular staining of PrP^sc ^in the neuropil with the rat monoclonal antibody R145 [37].

Histopathological examination of a haematoxylin-eosin stained section of the obex did not show vacuolar changes. IHC examination revealed PrP^sc ^accumulation in the spinal tract nucleus of the trigeminal nerve (fig. 1) in both cases, but not in the dorsal motor nucleus of the vagal nerve. PrP^sc ^was also demonstrated in the nucleus of the solitary tract in case 05/377. Both cases showed widespread PrP^sc ^immunolabelling of molecular, granular and white matter areas of the cerebellum.

**Figure 2:**
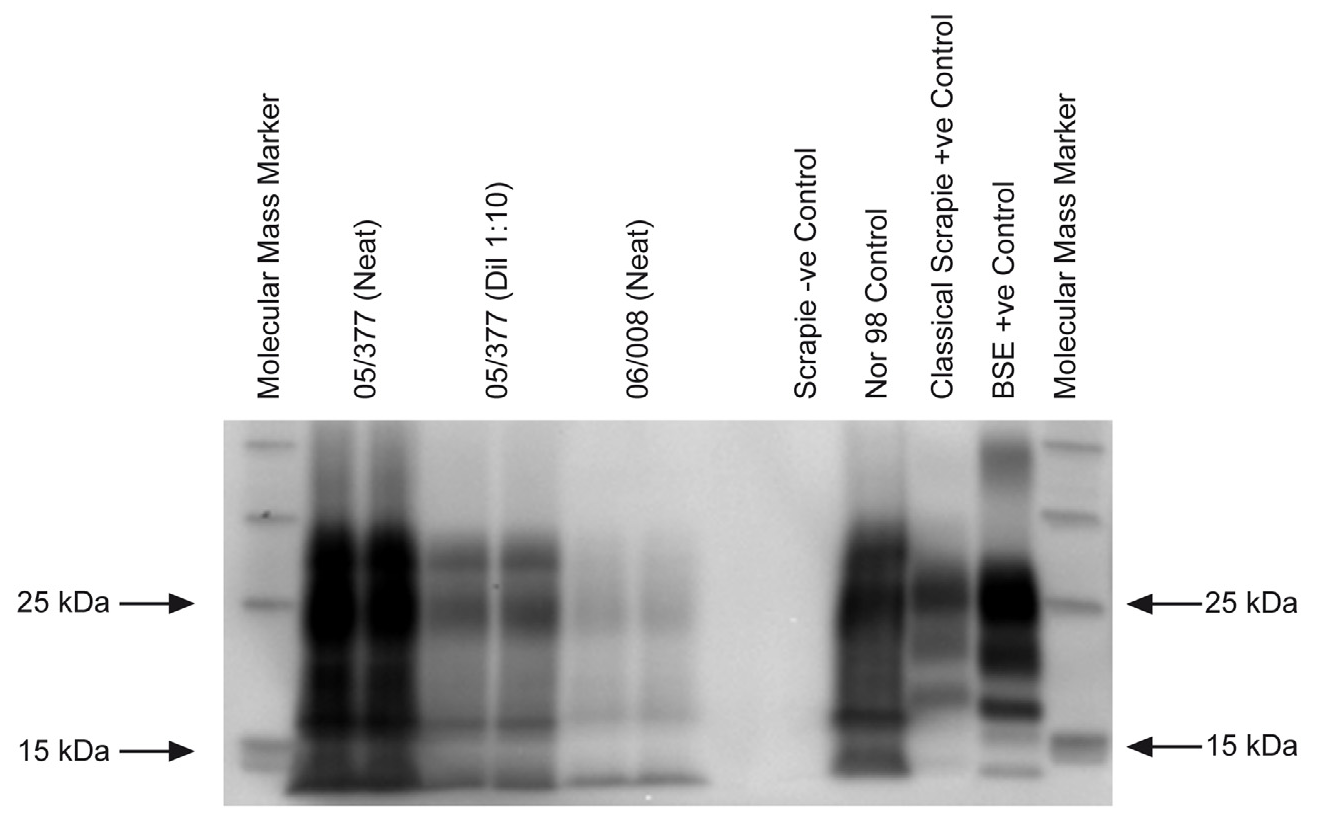
**Western immunoblot of cases 05/377 and 06/008**. Samples from 05/377 and 06/008 are shown in duplicate lanes; a diluted sample of case 05/377 was included to show the different bands more clearly. Controls are shown in single lanes and comprise a negative control sample (Suffolk, ARQ/ARQ), a known UK classical scrapie case (Poll Dorset, VRQ/VRQ), a known UK bovine BSE case and a known Nor98 Norwegian ovine scrapie case (Norwegian White, AHQ/AHQ). Molecular mass markers were included at either end of the gel (ECL DualVue, Amersham Biosciences, Little Chalfont, UK). The antibody used to detect PrP^res ^in the test kit is Sha31 [38].

The molecular profiles obtained using the TeSeE sheep/goat Western immunoblotting kit (Bio-Rad, Marnes-la-Coquette, France) on a fresh sample of the caudal brainstem from both 05/377 and 06/008 were unlike BSE and classical scrapie but similar to Nor98 with a band of molecular mass less than 15 kDa (fig. 2). Based on the IHC and molecular findings, the diagnosis of atypical scrapie was made.

## Discussion

A new form of scrapie, later termed atypical scrapie, has first been described in Norway in 2003 [5]. The diagnosis of atypical scrapie is made if the TeSeE sheep/goat Western blot reveals a recognisable protein band with a molecular mass of less than 15 kDa and conspicuous PrP^sc ^immunolabelling is detectable by IHC in the cerebellum and – at the obex level – in the nucleus of the spinal tract of the trigeminal nerve but absent in the dorsal motor nucleus of the vagal nerve [16]. Both cases fulfilled the criteria for atypical scrapie. In addition, both sheep were older sheep and carried the A F^141^RQ/AHQ PrP genotype, which has also been observed in other atypical scrapie cases [13,14,16].

Clinically, all affected animals in Norway reportedly displayed ataxia in the absence of pruritus; other infrequent signs were anxiety and loss of bodily condition [5]. The two cases reported as clinical suspects in Ireland all shared the same clinical sign, incoordination, in addition to weight loss and nervous behaviour [7]. The case in the Falkland Islands did not display any neurological signs when observed although the owner had reported collapsing episodes and persistent biting at its leg [8].

Clinical signs in classical scrapie can be very variable. Pruritus is a clinical sign usually associated with classical scrapie but its occurrence can vary significantly. In a recent study in Ireland, only 25% of 129 examined sheep displayed pruritus [2] whilst other studies in Italy and the UK reported pruritus in approximately 85% of 550 sheep [1] and 86% of 35 sheep [17] respectively. Ataxia was observed in 79.5% of 116 scrapie-affected sheep in Ireland and in 80% of sheep in the Italian study [1], whilst it was seen in all 35 sheep in the UK [17]. The reason for this variation is poorly understood but it may be the effect of genotype [2], breed or strain, which are thought to be the cause for the variation in the neuropathology in classical scrapie [18-20].

Both cases presented here were ataxic with general proprioceptive deficits, which suggests that incoordination may be a major clinical sign in atypical scrapie but, overall, the clinical signs were not identical although both sheep were of the same breed, genotype, age and born on the same farm, which is suggestive of infection with the same strain of agent. Both displayed signs suggestive of a cerebellar disease [21] but the signs were not identical (hypermetria in 05/377, ataxia, head tremor and abnormal menace response in 06/008). Although signs of pruritus, such as rubbing, scratching or nibbling parts of the body, were neither reported by the farmer nor observed at VLA, and large areas of alopecia were not present, case 05/377 displayed a positive scratch response, characterised by rhythmical head movements when the dorsum was scratched. A positive scratch response has been associated with teeth grinding and pruritus in classical scrapie [2]; teeth grinding was observed only once in case 05/377 during the observation and thus may not be significant. Case 05/377 was nervous whilst case 06/008 appeared to have a subdued mental status. A different clinical duration, which was not known for these cases, may be responsible for the different disease presentation. Indeed, the stronger signal obtained in case 05/377 by Western blot (fig. 2) suggests a higher level of PrP^res ^accumulation in the brainstem, which may indicate that this sheep was in a more advanced clinical stage. As the disease progresses scrapie cases are more likely to react to the scratch test, which was seen in case 05/377, but they also more frequently display head tremor, teeth grinding and an absent menace response [2,22], as seen in case 06/008, and argues against clinical duration being responsible for the observed differences.

It is still unknown which pathological features are responsible for the clinical signs observed in TSEs. Vacuolation in the brain is a common feature in classical scrapie [23] but the lack of vacuolation in atypical scrapie despite obvious signs of a neurological dysfunction [5] does not indicate that vacuolation is associated with neurological dysfunction. In addition, perikarya with vacuolation may appear otherwise unaltered histologically [24]. The cerebellar signs and behavioural abnormalities (nervousness, dullness suggestive of a forebrain disease, e.g. the frontal and temporal lobes [21]) observed in the two cases and other clinically affected sheep with atypical scrapie [5,7,25] appear to be associated with the PrP^sc ^accumulation in cerebellum and cerebral cortex as seen in atypical cases. However, whilst PrP^sc ^accumulation in the dorsal motor (parasympathetic) nucleus of the vagal nerve in cattle with bovine spongiform encephalopathy (BSE) [26] also seems to explain why this species displays bradycardia suggestive of an increased vagal tone [27], this is not a feature in classical scrapie [2,28] despite PrP^sc ^accumulation in the same nucleus [29]. Atypical cases generally present with PrP^sc ^accumulation in the spinal tract nucleus of the trigeminal nerve, which is involved in carrying pain and temperature sensations from the face [30]. Trigeminal neuralgia in humans causes paroxysmal pain confined to the trigeminal distribution, which may be provoked by light touch of the face although the clinical sensory examination is normal [31]. In an experimental model, the rat, trigeminal neuropathic pain caused hyper-responsiveness to stimulation of the injured nerve territory [32]. This was not observed in either sheep (see Additional file 4: Movie 4 showing no over-reaction to the palpebral reflex in case 06/008), and although the passive observation time was restricted to only 15 minutes there was also no evidence of excessive teeth grinding, which has been associated with trigeminal neuritis in cattle with BSE [33]. Thus, neurological dysfunction may be the result of other, not yet identified pathological processes.

The majority of atypical scrapie cases have been diagnosed as part of active surveillance in apparently healthy sheep or fallen stock [16], which indicates that the clinical presentation may be somewhat different to classical scrapie. In particular in the UK, where more than 4000 reported cases of scrapie in small ruminants have been diagnosed since 1993 [34], it would have been expected that farmers are familiar with the clinical signs and may be more likely to report sheep as clinical suspects. Indeed, a postal survey of the occurrence of scrapie in the UK conducted in 2002 has indicated that the degree of awareness of clinical signs of scrapie among farmers was high [35]. Both atypical cases here presented with signs suggestive of a neurological disease, such as scrapie. The Department for Environment, Food and Rural Affairs (Defra) published advisory notes for farmers in the UK [36] to aid in the identification of clinical suspects and advises to consider scrapie in any sheep or goat showing nervous signs or change in behaviour. This may explain why the first case on this farm (05/377) was reported as clinical suspect because of its history of changed temperament in combination with neurological signs. Abnormal behaviour was not reported for the second case (06/008) but the presence of an abnormal gait and previous experience of the farmer with the clinical signs in atypical scrapie may have led to the reporting of the second case.

## Conclusion

In summary, both cases exhibited signs of a neurological disease with similarities to classical scrapie. Incoordination, which may be expressed as ataxia with a crouching posture and wide-based stance or dysmetria, was observed in both atypical scrapie cases but there was considerable variation in the clinical presentation, which can only be evaluated if a clinical and neurological examination is performed.

Any sheep presenting with incoordination should be examined to investigate its cause, and if accompanied by other clinical signs associated with classical scrapie, such as loss of bodily condition, nervousness or dullness, tremor, an abnormal menace response or a positive scratch test, scrapie should be seriously considered.

## Authors' contributions

TK, AD and GB carried out the clinical assessments at VLA, JB provided the case history and assessed the sheep on the farm of origin, SE and MC were responsible for the Western immunoblot, GCS and SC were responsible for the sequencing of the PrP gene and MMS performed the neuropathological examination. All authors read and approved the final manuscript.

## Supplementary Material

Additional File 1 "Positive scratch test in classical scrapie". Welsh mountain ewe (ARQ/VRQ), 3 years of age, responding to scratching of the femur with lip movements ("nibble reflex"). Click here for file

Additional File 2 "Hypermetria in case 05/377". Welsh Mountain ewe displaying hind limb hypermetria. Click here for file

Additional File 3 "Ataxia in case 06/008". Welsh Mountain ewe displaying hind limb ataxia with crouching on turns. Click here for file

Additional File 4 "Absent menace response in case 06/008". Welsh Mountain ewe with an absent menace response but normal blink reflex suggestive of a cerebellar dysfunction (vision unaffected, see movie 3, intact sensory branch of the trigeminal nerve, intact facial nerve). Click here for file

Additional File 5 "Negative scratch test in case 06/008". Welsh Mountain ewe, which does not respond to scratching of the dorsum, shoulder or femur. Click here for file
